# Editorial: Insights in AI: Medicine and public health 2022

**DOI:** 10.3389/frai.2023.1166426

**Published:** 2023-05-02

**Authors:** Thomas Hartung, Jun Deng, Raghvendra Mall, Alejandro F. Frangi, Frank Emmert-Streib, Tuan D. Pham

**Affiliations:** ^1^Bloomberg School of Public Health, Johns Hopkins University, Baltimore, MD, United States; ^2^Department of Therapeutic Radiology, Yale University, New Haven, CT, United States; ^3^Biotechnology Research Center, Technology Innovation Institute, Masdar City, United Arab Emirates; ^4^University of Leeds, Leeds, United Kingdom; ^5^Predictive Society and Data Analytics Lab, Tampere University of Technology, Tampere, Finland; ^6^Center for Artificial Intelligence, Prince Mohammad Bin Fahd University, Khobar, Saudi Arabia

**Keywords:** AI, precision medicine, precision health, machine learning, oncology, COVID-19, pharmacogenomics

A recent report on the 100-year study of AI at Stanford University (Littman et al., 2021) claimed that one of the two most promising opportunities for AI is its power to augment human knowledge and skills, where health and medicine made a leading story: “For example, an AI system may be able to synthesize large amounts of clinical data to identify a set of treatments for a particular patient along with likely side effects; a human clinician may be able to work with the patient to identify which option best fits their lifestyle and goals, and to explore creative ways of mitigating side effects that were not part of the AI's design space.” These capacities are referred to as personalized or precision health (Genomics and Precision Health, 2022).

After recovering from setbacks of the “AI winter” during the periods of 1974–1980 and 1987–1994 (Lutkevich, 2022), applications of AI have been pervading most aspects of human life at accelerating rates. The impacts are largely due to the development of “deep learning” models in machine learning (a subset of AI), including convolutional and recurrent neural networks, together with the advancement of computing hardware.

Pharmacogenomics, precision oncology, and electronic health records (EHR) are typical areas that are promising for delivering precision medicine and precision health. The aim of pharmacogenomics is to identify how a patient's DNA responds to certain drugs (Shin et al., 2009). Instead of using the “one size fits all” assumption, precision oncology can recommend targeted therapy by studying the molecular characteristics of a patient's tumor (Hodson, 2020). EHR of patients are of essential information for modern healthcare that can be utilized to enhance patient care, reduce hospital costs, provide resources for clinical research, develop efficient healthcare management, etc. (Johnson and Stead, 2022). Breakthroughs in these fields would be hardly achieved without relying on the state-of-the-art AI.

*Insights in AI: Medicine and public health* aimed to timely collect high-quality research works to reflect current developments on AI-enabled medicine and healthcare. This Research Topic consists of six peer-reviewed articles contributed by scientists working across interdisciplinary areas of health, medicine, and computing.

“*Statistical biopsy: An emerging screening approach for early detection of cancers*” by Hart et al. trained neural networks with personal health data for risk prediction in several cancer types. The proposed model was supported with results obtained from using the UK Biobank data for testing.

“*Pharmacogenomics driven decision support prototype with machine learning: A framework for improving patient care*” by Kidwai-Khan et al. used several machine-learning models to learn on EHR and genetic data for pharmacogenomics-based prediction of medications at the point of care in near real time. Results obtained in terms of two statistical measures of performance suggested the effectiveness of the proposed prototype.

Another paper entitled “*Interpretable machine learning for predicting pathologic complete response in patients treated with chemoradiation therapy for rectal adenocarcinoma*” by Wang et al. applied the so-called “explainable boosting machine” model to learn on clinical parameters and features extracted from CT images for identifying rectal-cancer patients who may not need surgery after neoadjuvant chemoradiotherapy. In particular, the proposed approach was able to discover distinct features associated with poor outcomes.

AI plays an important role for the prevention and control of COVID-19 disease. The article entitled “*A survey of COVID-19 detection and prediction approaches using mobile devices, AI, and telemedicine*” by Shen et al. surveyed current research and development on connected mobile devices through the internet of things and AI for COVID-19 detection, monitoring before and after infection, and contact tracing. Current challenges were also discussed, particularly the issue of data privacy.

In addition to the importance of EHR, electronic dental records were used for machine learning to differentiate between healthy subjects and patients with periodontal disease (PD), and severity levels of PD. This work was reported in the paper entitled “*Developing and testing a prediction model for periodontal disease using machine learning and big electronic dental record data*” by Patel et al.

Finally, “*Artificial intelligence assisted acute patient journey*” by Nazir et al. conducted a mini-review on the emerging shift in healthcare systems *via* the implementation of AI and digital transformation toward cyber-physical domain, which contributes to the reality of precision healthcare.

We hope this Research Topic provides its readers with insights into how AI can significantly enhance medicine and public health for better wellbeing.

## Author contributions

All authors listed have made a substantial, direct, and intellectual contribution to the work and approved it for publication.
